# Retinal Protection by Sustained Nanoparticle Delivery of Oncostatin M and Ciliary Neurotrophic Factor Into Rodent Models of Retinal Degeneration

**DOI:** 10.1167/tvst.10.9.6

**Published:** 2021-08-04

**Authors:** Jing-Yan Yang, Bin Lu, Qiang Feng, Jorge S. Alfaro, Po-Hsuen Chen, Joseph Loscalzo, Wen-Bin Wei, Ying-Yi Zhang, Shi-Jiang Lu, Shaomei Wang

**Affiliations:** 1 Regenerative Medicine Institute, Cedars-Sinai Medical Center, Los Angeles, CA, USA; 2 NanoNeuron Therapeutics and HebeCell Corp., Natick, MA, USA; 3 Department of Medicine, Brigham and Women's Hospital, Harvard Medical School, Boston, MA, USA; 4 Beijing Tongren Hospital, Capital Medical University, Beijing, P.R. China

**Keywords:** neuroprotection, trophic factors, intravitreal drug delivery

## Abstract

**Purpose:**

Retinitis pigmentosa (RP) is caused by mutations in more than 60 genes. Mutation-independent approaches to its treatment by exogeneous administration of neurotrophic factors that will preserve existing retinal anatomy and visual function are a rational strategy. Ciliary neurotrophic factor (CNTF) and oncostatin M (OSM) are two potent survival factors for neurons. However, growth factors degrade rapidly if administered directly. A sustained delivery of growth factors is required for translating their potential therapeutic benefit into patients.

**Methods:**

Stable and biocompatible nanoparticles (NP) that incorporated with CNTF and OSM (CNTF- and OSM-NP) were formulated. Both NP-trophic factors were tested in vitro using photoreceptor progenitor cells (PPC) and retinal ganglion progenitor cells (RGPC) derived from induced pluripotent stem cells and in vivo using an optic nerve crush model for glaucoma and the Royal College of Surgeons rat, model of RP (*n* = 8/treatment) by intravitreal delivery. Efficacy was evaluated by electroretinography and optokinetic response. Retinal histology and a whole mount analysis were performed at the end of experiments.

**Results:**

Significant prosurvival and pro-proliferation effects of both complexes were observed in both photoreceptor progenitor cells and RGPC in vitro. Importantly, significant RGC survival and preservation of vision and photoreceptors in both complex-treated animals were observed compared with control groups.

**Conclusions:**

These results demonstrate that NP-trophic factors are neuroprotective both in vitro and in vivo. A single intravitreal delivery of both NP-trophic factors offered neuroprotection in animal models of retinal degeneration.

**Translational Relevance:**

Sustained nanoparticle delivery of neurotrophic factors may offer beneficial effects in slowing down progressive retinal degenerative conditions, including retinitis pigmentosa, age-related macular degeneration, and glaucoma.

## Introduction

Retinitis pigmentosa (RP), a heterogeneous group of inherited retinal degenerative disorders, affects 1 in 3500 to 4000 people.^1^^–^^4^ There are diverse genetic causes of RP, including up to 3000 different mutations in more than 60 genes with dominant, recessive, and sex-linked inheritance patterns.^5^^–^^7^ The most common feature of RP is a gradual breakdown of rods (detecting dim light) and cones (detecting light and color). RP starts with night blindness and eventually loss of peripheral vision, with only central vision remaining (so-called tunnel vision). As the degeneration progresses, patients lose central vision and become blind. Current treatments for RP are very limited.^1^^,^^8^^–^^10^ RPE65 gene replacement therapy has received approval from the US Food and Drug Administration as a treatment for Leber congenital amaurosis, comprising a small percentage of all known RP.^11^^–^^14^ Yet, although such mutation-specific approach for RP is ideal, the extensive genetic heterogeneity renders it impractical for clinical application. Preserving the existing retinal anatomy and visual function, regardless of the specific mutations such as through neuroprotection, is a rational strategy.

Previous studies have demonstrated that neurotrophic factors can slow the rate of the progression of retinal degenerations.^15^^,^^16^ Of these factors, ciliary neurotrophic factor (CNTF), expressed by Müller and retinal pigment epithelium (RPE) cells, has shown a promising capability to promote photoreceptor and retinal ganglion cell (RGC) survival in a variety of animal models.^17^^–^^22^ More important, clinical trials in patients with RP and AMD showed that CNTF treatment preserved retinal thickness and slowed down the progression of vision loss, as determined by electroretinography (ERG).^21^^,^^23^ Oncostatin M (OSM), another neurotrophic factor and member of the IL-6 family of cytokines, promotes a variety of biological effects, including inhibiting neurodegeneration and excitotoxic injury induced by the expression of IL-6 and other cytokines in the central nerves system.^24^ Additionally, OSM has been shown to protect RGCs in an optic nerve crush (ONC) model and photoreceptors in a rodent model of recessive RP in short-term studies.^25^^,^^26^

Several methods have been employed for intraocular delivery of neurotrophic factors. These methods include direct injections of protein,^25^^,^^27^ injections of an adeno-associated virus vector that encodes the specific protein,^19^^,^^28^^–^^30^ and implantation of encapsulated cells that secrete the selected protein.^21^^,^^23^^,^^31^ In the current study, we examined an alternative method that uses a polysaccharide nanoparticle (NP) as a carrier for the long-term release of neurotrophic factors. Two animal models were used; one is the ONC model of acute glaucoma-induced retinal injury, and the Royal College Surgeon (RCS) rat, a well-established model of recessive RP. ONC has been used to test several therapeutic interventions.^32^^–^^36^ The primary defect in the RCS rat is caused by a loss-of-function mutation in the c-mer proto-oncogene tyrosine kinase (MERTK) gene, which was established as a human disease gene by the identification of disease-associated mutations in individuals with a form of autosomal recessive RP.^37^ The mutation in MERTK gene carried by the RCS rats disrupts the ability of RPE cells to phagocytose photoreceptor outer segments. The undigested photoreceptor outer segments accumulate as toxic debris in the subretinal space between the outer nuclear layer (ONL) and the RPE, which blocks nutrient access to photoreceptors from the choroid vasculature, leading to progressive photoreceptor death and commensurate visual loss.^38^^,^^39^

In this study, we demonstrated that a single intravitreal injection of CNTF-NP or OSM-NP protected RGCs in an acute glaucoma model, as well as photoreceptors and vision for more than 70 days in a rat model of RP. This approach holds the potential to translate into the clinic as a viable retinal neural protection strategy.

## Methods

### Preparation of CNTF-NP and OSM-NP

**Figure 1.**
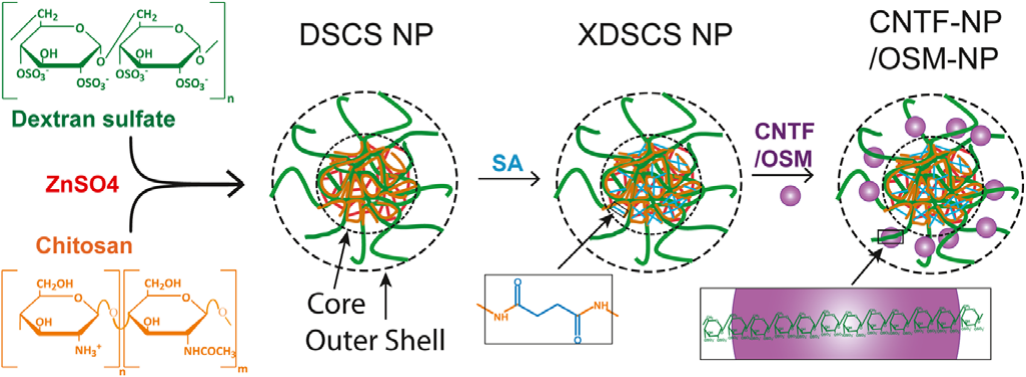
Diagram of NP preparation and CNTF-NP and OSM-NP formulation. Complexing DS and chitosan (CS) led to the formation of DSCS NP, which contains a charge-neutralized hydrophobic core and a negatively charged outer shell comprising DS. The core of the particle was subsequently crosslinked with succinic acid, resulting in XDSCS NP. Neurotrophic factors, CNTF or OSM, were loaded on the outer shell of XDSCS NP via their heparin-binding domains complexed with DS, giving rise to CNTF- or OSM-NP.

Polysaccharide NP were prepared in three stages (Fig. 1), resulting in DSCS NP, XDSCS NP, and CNTF- and OSM-NP. DSCS NP were prepared according to a previously described method.^40^ To increase the stability of DSCS NP in salt solutions, XDSCS NP were prepared by covalently crosslinking chitosan with succinic acid within the core of DSCS NP. DSCS NP was suspended in 100 mM HEPES buffer (pH 7.0), mixed with succinic acid (20 mM) for 1 hour, and centrifuged (at 15,000×*g* for 15 minutes). The concentrated precipitated particles were resuspended in 100 mM HEPES buffer, added with coupling reagents, EDC and NHS (45 mM each) added, and mixed for 20 hours at room temperature. The resulting particles were concentrated by centrifugation, and incubation in 3× DPBS for 2 hours, and removing large aggregates via low-speed centrifugation (200×*g* for 15 minutes). The XDSCS NP were subsequently washed with DPBS, filtered through an 0.22-µm PVDF membrane, precipitated, and resuspend in 5% mannitol for storage at −80 °C, to which CNTF- or OSM-NP, XDSCS NPs were diluted in DPBS and were slowly added (approximately 0.2 mL/min) with CNTF (Novoprotein #C099) or OSM (Bonopus # BP07001C) while stirring at 800 rpm. After 20 minutes of mixing, unincorporated protein was separated from NP-incorporated protein by centrifugation. SDS PAGE and densitometry analyses were performed to estimate the incorporation efficiency. CNTF-NP or OSM-NP were suspended in 5% mannitol at approximately 1 mg (protein)/mL and stored at –80 ^○^C until use.

The diameter and zeta potential of the polysaccharide NP were characterized by dynamic light scattering method using a Zetasizer from Malvern Instruments)(Malvern, UK) according to the manufacturer's instruction. The amount of un-neutralized DS in the particles was measured by Azure A assay, from which the un-neutralized glucose sulfate units in the particles were calculated.^40^ Incorporation efficiencies of CNTF or OSM into xNPs were determined by SDS gel electrophoresis followed by densitometry analysis using methods as previously described.^40^

### Generation of Photoreceptor and Retinal Ganglion Progenitor Cells (RGPCs)

Three induced pluripotent stem cell (iPSC) lines used in this study were generated from human dermal fibroblast cells using mRNA reprograming technology^41^ (the StemRNA-NM Reprogramming kit, Stemgent). The iPSCs were induced to differentiate into photoreceptor progenitor cells (PPC) sequentially by exposure to different media. Briefly, neural induction was initiated by a neural induction medium consisting of DMEM/F12 supplemented with 1% N2 and 1% B27, 20 µg/mL insulin (Roche, Basel, Switzerland) and 100 ng/mL Noggin (Peprotech, East Windsor, NJ) for 14 days. PAX6^+^ cells (95%) were transferred into ultra-low attachment dishes in neural differentiation medium consisting of neural induction medium without noggin and insulin to form neurospheres for 5 days. Subsequently, the formed neurospheres were replated on laminin-511–coated dishes for 35 to 40 days with one-half volume media changed every 2 to 3 days. At day 60 (D60) cells were then replated on ultra-low attachment dishes to facilitate formation of neurospheres for 2 to 3 days, and neurospheres were cryopreserved using Cryostem medium (Biological Industries, Beit HaEmek, Israel) for future use.

For induction of RGPC, D14 PAX6^+^ cells were switched to neural ganglion medium consisting of neurobasal medium (Thermo Fisher Scientific, Waltham, MA) supplemented with 5 µM forskolin and 10 ng/mL brain-derived neurotrophic factor. Cultures were continued as a monolayer for another 5 to 6 days with one-half medium changed every 2 days. At about day 20, cells were transferred into low-attachment dishes to form neurospheres in neural ganglion medium for 3 to 5 days. Neurospheres were then plated on Matrigel-coated dishes and maintained for another 14 days. At day 35 (D35) RGPC were cryopreserved using Cryostem freezing medium (Biological Industries) and banked for future usage.

### Functional Assay of Neurotrophic Factors In Vitro

At D60, photoreceptor progenitor neurospheres were thawed in neural differentiation medium media, dissociated with TripLE (Thermo Fisher Scientific) into single cells, and seeded on Matrigel-coated 24-well plates at a density of 5 × 10^4^ cells/well. Similarly, at D35 retinal ganglion progenitor neurospheres were dissociated with TripLE into single cell, resuspended in Neural Ganglion Medium, and seeded in Matrigel-coated 24-well plates at a density of 5 × 10^4^ cells/well. Treatment with CNTF, CNTF-NP, OSM, or OSM-NP was initiated 48 hours after seeding. Culture media were refreshed every 3 to 4 days while maintaining the same concentration of proteins in the media. Treatments were performed in triplicate and experiments were repeated at least twice. At 30 days after treatment, cells were collected, and viable cells were counted.

### Animals and Experimental Designs

The RCS rats were originally purchased from the Rat Resource & Research Center and maintained in our breeding colony. Long Evans rats (8–10 weeks old, purchased from Charles River Laboratories, Wilmington, MA) were maintained in our breeding colony. Animals were housed at the Cedars-Sinai Medical Center Department of Comparative Medicine vivarium. All animal protocols were approved by the Institutional Animal Care and Use Committee and all animals were treated in accordance with the ARVO Statement for the Use of Animals in Ophthalmic and Vision Research. RCS rats received a unilateral single intravitreal injection of CNTF-NP, OSM-NP, and phosphate-buffered saline (PBS) alone at postnatal day (P) 21 to 23; the fellow eyes served as untreated controls.

#### ONC Model and Intravitreal Injection

ONC in Long Evans rats was performed following our published protocol.^42^ Complete ONC was verified by demonstrating no pupillary light reflex after lesion creation. Immediately after ONC, 4 µL of OSM-NP, CNTF-NP, or PBS alone were intravitreally injected. For each intravitreal injection, the micropipette was inserted in the peripheral retina, just behind the ora serrata, and was angled to avoid damage to the lens; the fellow eyes served as untreated controls.

#### Visual Function Test

All animals were tested by ERG and optokinetic response (OKR), according to our previous studies.^43^^,^^44^ OKR is a noninvasive method to evaluate visual acuity quantitively. ERG provides a gross measure of retinal response to light stimuli. RCS rats were tested at two time points (P60 and P90) before being sacrificed for histology.

#### Histology

Animals were euthanized at the end of experiment. Eyes were collected and processed for cryostat sections (10 µm) as described in our previous studies.^43^^,^^45^ Four sections/slide were collected in five series and stored at −80 °C. One slide from each series was stained with cresyl violet to assess the integrity of retinal lamination. Other slides were used for antibody staining, following previously described protocols, and were examined by regular light and confocal microscopy (Eclipse C1si; Nikon Instruments, Inc., Melville, NY). The following antibodies were used: CNTF (rabbit polyclonal, 1:500, Santa Cruz Biotechnology, Dallas, TX), Cone-arrestin (rabbit polyclonal, 1:1,000; Millipore, Burlington, MA), and protein kinase C-alpha (rabbit polyclonal, 1:5000; Sigma, St Louis, MO). Anti-rabbit secondary antibodies conjugated to Alexa Fluor-488 (Life Technologies, Carlsbad, CA) were used and sections were counterstained with 4′, 6-diamidino-2-phenylindole.

#### Retinal Whole Mount Preparation

Eyes were fixed in 4% paraformaldehyde in 0.01 M PBS (pH 7.4). A retinal whole mount was prepared and stained with goat Brn-3a antibody (1:200) following our previous protocol.^46^^,^^47^ Retinal whole mount was examined by fluorescence microscopy.

#### Quantitative Analysis and Statistical Analysis

For the retinal whole mount preparation (*n* = 8), a total of 12 images per retina (three images from each retinal quantum) were taken under fluorescent microscopy. Brn-3a–positive cells were counted using ImageJ (NIH, Bethesda, MD) and analyzed by two-way analysis of variance (ANOVA) with Bonferroni post hoc power analyses (GraphPad Prism 9 statistical analysis software). *P* values of 0.05 or less were considered significant. Error bars indicate standard error of the mean. For ONL protection in the RCS rats, the length of the ONL with more than two cell thicknesses was measured using the ImageJ program and compared with the whole retinal length on transverse section (presented as a percentage of protection, 12 sections per retina; *n* = 6 per treatment). The control treatment was not included because there is only one cell thickness of the ONL remaining.

Visual acuity and b-wave amplitudes of CNTF-NP, OSM-NP, and PBS treated eyes or untreated eyes (*n* = 8 rats per group) were analyzed by two-way ANOVA with Bonferroni post hoc power analysis (GraphPad Prism 9 statistical analysis software). *P* values of 0.05 or less were considered significant. Error bars indicate standard error of the mean.

## Results

### Formulation of CNTF-NP and OSM-NP

The procedure for CNTF- and OSM-NP preparation is described under Methods and outlined in Figure 1. The particles’ physical parameters are shown in Table. Because CNTF and OSM are heparin-binding proteins,^48^^,^^49^ and dextran sulfate (DS) is an analog of heparin, incorporation of CNTF or OSM to XDSCS NP occurred rapidly. The optimal ratio for CNTF incorporation was found to be 0.35:1 (mg/mg, CNTF/DS), at which 91 ± 5% of the input CNTF was bound to XDSCS NP without causing aggregation (Table). For OSM incorporation, the optimal ratio was 0.5:1 (mg/mg, OSM/DS), at which ration 92 ± 5% of the input OSM was incorporated.

**Table.** Physical Properties of Particles Formed at Each Stage of Preparation^*^

	Diameter (nm)	Polydispersity	Zeta Potential (−mV)
DSCS NP	417 ± 12	0.079 ± 0.014	−46.4 ± 3.6
xNP	371 ± 14	0.041 ± 0.021	−50.0 ± 4.7
CNTF NP	338 ± 4	0.068 ± 0.023	−39.8 ± 1.3
OSM NP	317 ± 3	0.042 ± 0.015	−38.9 ± 0.7

* Data are obtained from 10 to 12 separate preparations and presented as mean ± standard error of the mean.

#### Activities on Cultured Retinal Progenitor Cells

**Figure 2.**
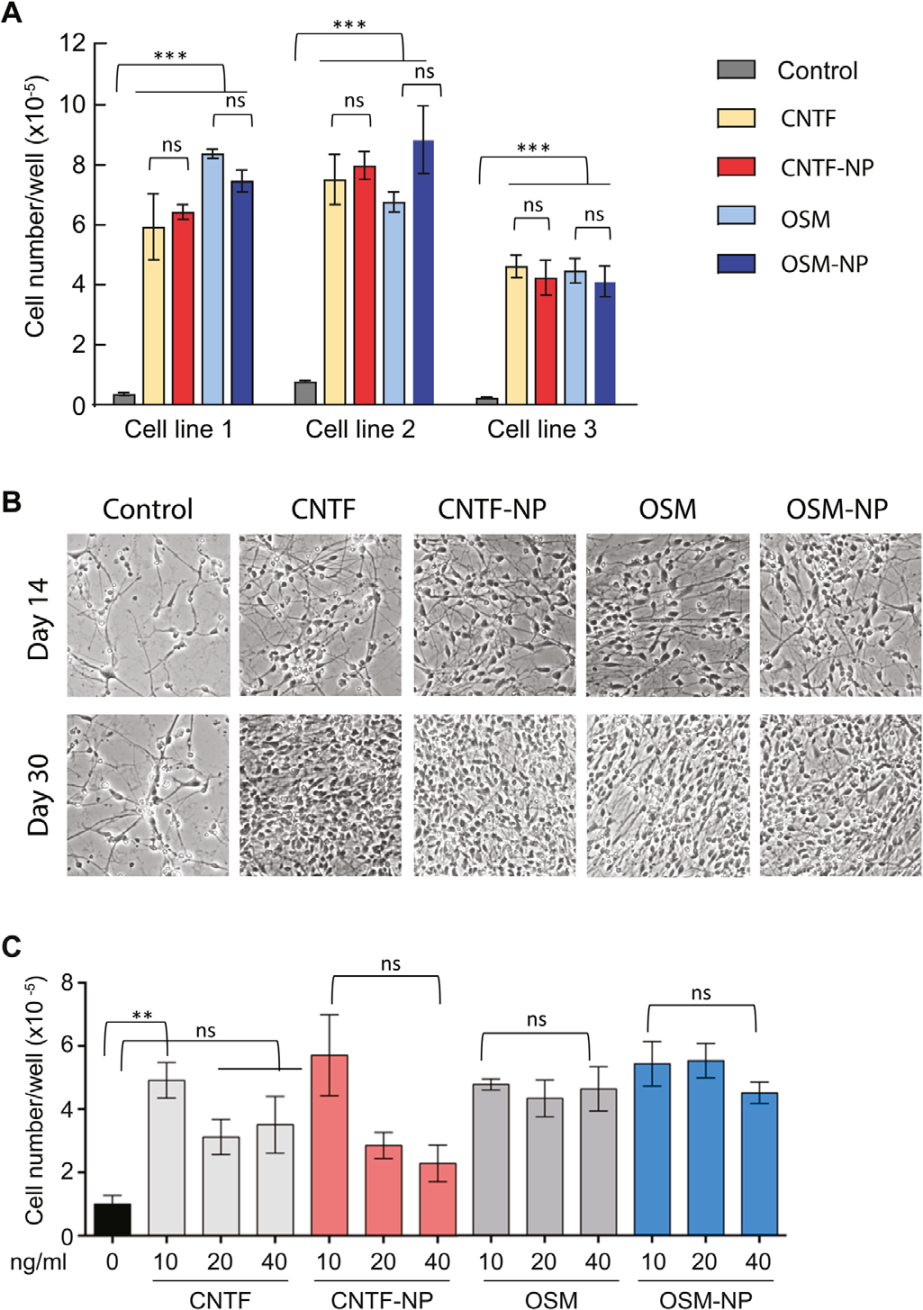
Effects of free and NP-bound CNTF and OSM on retinal progenitor cell proliferation. (A) PPC were derived from three human iPS cell lines and cultured in Neural Differentiation Medium with or without 10 ng/mL CNTF, CNTF-NP, OSM, or OSM-NP. Culture media were refreshed every 3 to 4 days, and viable cells were counted after 30 days of the culture. All the three cell lines tested showed significant difference among trophic factors or NP-trophic factors over control (*P* < 0.001), while the difference among trophic factors and NP-trophic factors (*P* > 0.05). (B) Representative of PPC cultured under conditions as that described in (A). (C) Human iPS cell-derived RGPC were cultured in neural ganglion medium in the absence or presence of indicated forms of CNTF and OSM at concentrations of 10, 20, or 40 ng/ml. Viable cells were counted at day 30 of the culture. The difference between CNTF or CNTF-NP over control was significant (*P* < 0.01); higher concentration of CNTF or CNTF-NP did not reach significant difference over control (*P* > 0.05). However, the difference between OSM or OSM-NP at all the three concentration over control was significant (*P* < 0.01), while the difference among the three concentration of OSM or OSM-NP was not significant (*P* > 0.05). Data were obtained from three separate experiments and presented as mean ± standard error of the mean. Two-way ANOVA, Newman–Keuls multiple comparison. ** *P* < 0.01, *** *P* < 0.001.

Neurotrophic activities of free and NP-incorporated CNTF and OSM were examined in cultured PPC and RGPC. As shown in Figure 2A, the proliferation of PPC in the control medium was minimal (cell number increased by <2-fold after a 30-day culture) but was markedly enhanced (8- to 16-fold) with the supplemental of 10 ng/mL CNTF, CNTF-NP, OSM, or OSM-NP. Morphologically, the control cultures displayed a scattered population with limited cell–cell connections, whereas the treated cultures showed a densely packed network of neural connections associated with an abundance of cell bodies with a “healthy” appearance (Fig. 2B). CNTF and OSM in both free and NP-incorporated forms exerted the same degree of the pro-proliferation effect. In the RGPC culture (Fig. 2C), supplemental of 10 ng/mL CNTF, CNTF-NP, OSM, or OSM-NP promoted the cell proliferation by approximately five-fold as compared with that of control. At higher concentrations (20 and 40 ng/mL), the effect of CNTF was decreased, but that of OSM remained unchanged. CNTF-NP and OSM-NP showed the same pattern of effects as that of their free forms. These data indicated that the NP-bound CNTF and OSM exerted the comparable neurotrophic activity as that of free CNTF and OSM.

### Efficacy After Single Intravitreal Delivery of CNTF-NP or OSM-NP Into the ONC Rat Model for Glaucoma

**Figure 3.**
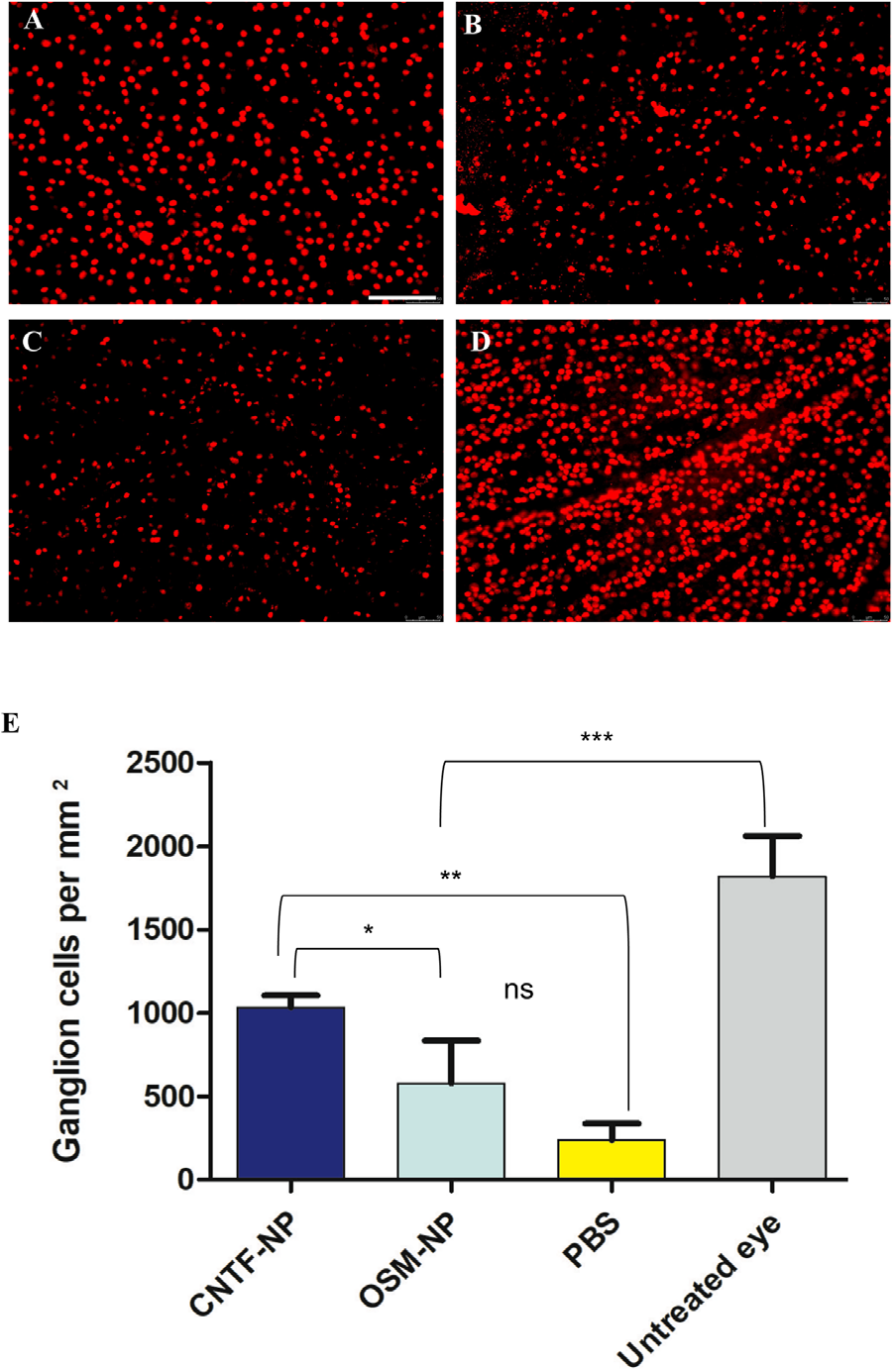
Effect of NP-bound CNTF and OSM on RGC protection in an ONC model of glaucoma. Retinal whole mount stained with the RGC marker, Brn3a, 2 weeks after ONC (*n* = 8/treatment). Significantly higher RGC density was observed in NP-CNTF- treated retina (A) compared with NP-OSM- (B) and PBS-treated eyes (C), there is a significantly RGC loss following ONC compared with the untreated fellow eye (D) (Scale bar = 100 µm). Two-way ANOVA, Newman–Keuls multiple comparison . * *P* < 0.05, ** *P* < 0.01, *** *P* < 0.001.

The in vitro study showed both CNTF-NP and OSM-NP promoted survival and proliferation of iPSC-derived retinal progenitor cells. To investigate further their neuroprotective effect in vivo, both NP complexes were tested in a well-established ONC model of glaucoma. Figure 3 shows representative fluorescence images of OSM-NP, CNTF-NP, and PBS alone treated retinas (Figs. 3A–C) and nonoperated control (Fig. 3D). A single intravitreal injection of either CNTF-NP or OSM-NP promoted RGC survival compared with PBS-treated controls (Figs. 3A and B vs. Fig. 3C). In the control eye, the number of RGCs was significantly decreased 2 weeks after ONC, compared with that of the intact, nonoperated eye (Fig. 3C vs. Fig. 3D), whereas in the NP-trophic factor-treated eyes, there was greater RGC survival (Figs. 3A and B vs. Fig. 3C). Quantification of RGC counts (Fig. 3E) revealed the mean RGC densities in nonoperated eyes were significantly higher than that in other groups (*P* < 0.001), CNTF-NP–treated eyes had significantly higher RGC densities compared with OSM-NP–treated and PBS-treated groups (*P* < 0.05); the OSM-NP–treated eyes had more RGC survival than PBS treated eyes, but the difference was not significant (*P* > 0.05) (two-way ANOVA, Newman–Keuls multiple comparison).

### Efficacy After Single Intravitreal Delivery of CNTF-NP or OSM-NP Into the RCS Rat Model of RP

**Figure 4.**
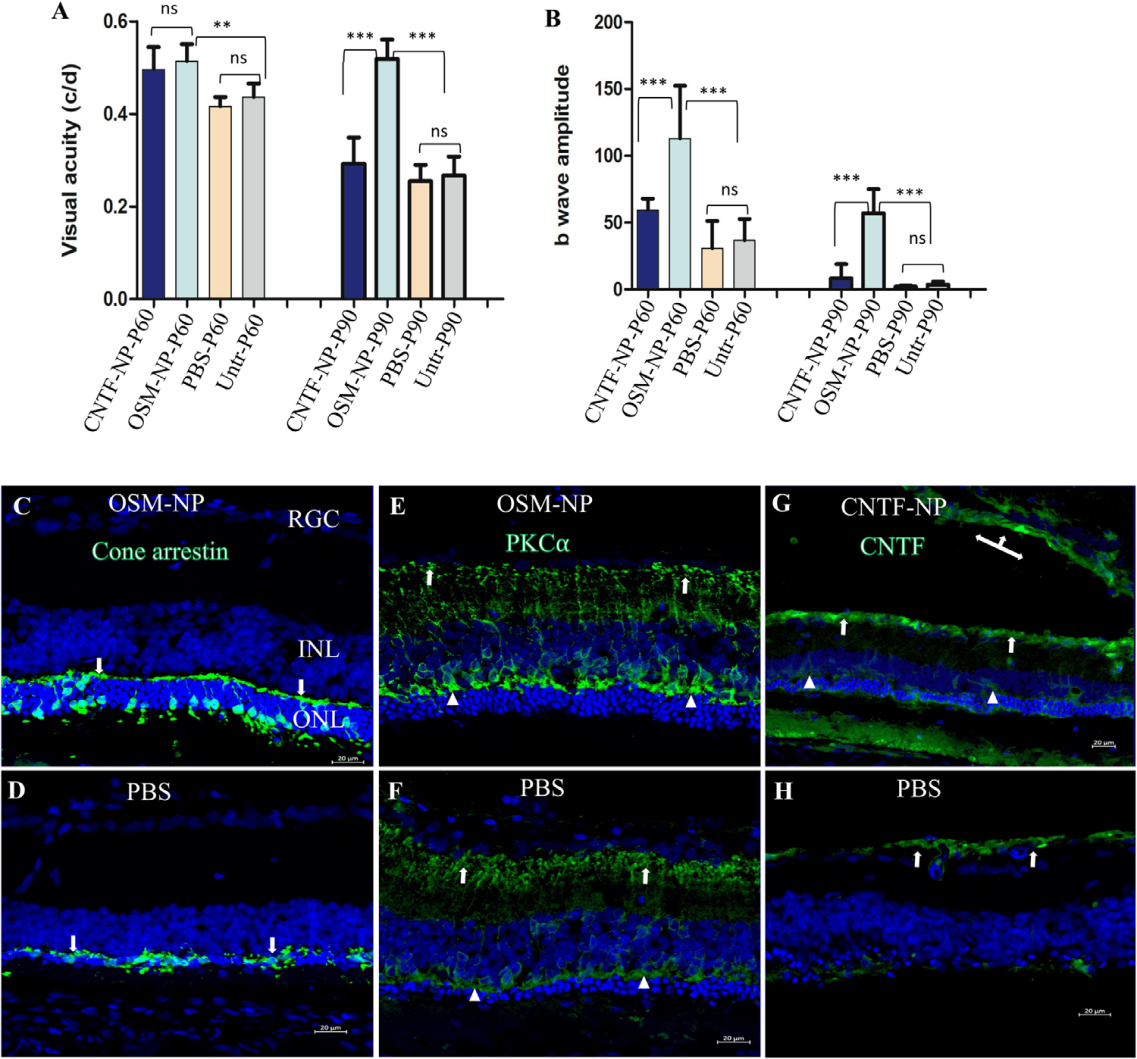
Effect of CNTF-NP and OSM-NP on photoreceptor and vision protection in a RP model. A single intravitreal injection of CNTF-NP, OSM-NP, and PBS into RCS rats, model of RP (*n* = 6/treatment) at postnatal day (P) 21 to 23, efficacy was assessed by testing OKR and ERG at P60 and P90 time points. (A) Visual acuity revealed by OKR shows both CNTF-NP and OSM-NP treated groups are significantly higher than controls at P60 (*P* < 0.01); however, only the OSM-NP–treated group has a significantly higher visual acuity than other three groups and remains unchanged at P90 (*P* < 0.001). The difference between CNTF-NP and OSM-NP treated groups is not significant at P60, and there is no difference between PBS-treated and untreated controls at both P60 and P90 time points tested. (B) ERG shows that only the OSM-NP treated group has significantly higher b-wave than other three groups (*P* < 0.001) at both time points tested, although b-wave amplitude is decreased at P90 compared with that at P60. The CNTF-NP–treated group has higher b-wave than the controls, but the difference is not significant (*P* > 0.05). Two-way ANOVA, Newman–Keuls multiple comparison . ** *P* < 0.01, *** *P* < 0.001. Cryostat sections from OSM-NP–treated eyes stained with cone arrestin antibody showed preserved cone morphology with cell bodies, pedicles (arrows in C), as well as some segments (C), although the typical cone morphology was absent in control retinas (arrows in D). Retinal secondary neuron–rod bipolar cells as revealed by protein kinase C-alpha antibody had greater axonal (arrows in E) and dendritic (triangles) terminal density in OSM-NP–injected eyes, compared with controls (arrows in F). The immunostaining revealed by anti-CNTF antibody indicated a higher expression of CNTF in the RGC layer (arrows in G) and Muller glia (triangles in G) in CNTF-NP–treated eyes compared with control (H), where the weak positive staining was restricted to the RGC layer (arrows in H). It is noted that a strong CNTF staining was also seen in the vitreous (left-right-up arrows in G). Abbreviations: INL, inner nuclear layer; ONL, outer nuclear layer; RGC, retinal ganglion cell; Untr, untreated.

The RCS rat is a well-established model of RP. In the untreated RCS rat, only a single layer of photoreceptors remains at P90. Here, photoreceptor survival and visual function were assessed in the RCS rats that received intravitreal injection of OSM-NP, CNTF-NP, PBS, or no treatment. Spatial visual acuity in units of cycle/degree assessed by OKR is significantly higher in CNTF-NP–treated and OSM-NP–treated rats at P60 compared with controls (*P* < 0.01) (Fig. 4A). There is no difference between CNTF-NP– and OSM-NP–treated groups at this time point. However, only the OSM-NP–treated group has significant difference over other three groups (*P* < 0.001) at P90. The difference between PBS-treated and untreated controls is not significant at either the P60 or the P90 time points tested. In addition, retinal responses to light stimulation were measured by ERG. Here, b-wave amplitudes after OSM-NP injections are significantly higher when compared with other three groups at both P60 and P90 (*P* < 0.001), whereas CNTF-NP injection has higher b-wave then the controls at P60; however, the difference is not significant (*P* > 0.05) (Fig. 4B).

To investigate whether OSM-NP and CNTF-NP preserved photoreceptors, we applied cresyl violet staining on frozen retinal transverse sections from RCS rats with different treatments. Retinal sections showed clear protection of photoreceptors in OSM-NP–treated eyes observed at P90 with an ONL thickness of four to five nuclei in comparison with CNTF-NP–treated eyes (two to three nuclei) and untreated eyes with just one row of photoreceptors remaining. To quantify ONL preservation, the length of ONL with more than two cell thicknesses was measured with the ImageJ program against the whole retinal length on transverse section. Quantification was done only on treated eyes, because only a single layer of ONL remained in the control eye. The quantification of ONL preservation showed 37.62 ± 8.79% of photoreceptor protection in OSM-NP–treated eyes and 19.72 ± 7.2% in CNTF-NP–treated eyes.

To study further photoreceptors and inner retinal preservation, retinal sections were stained with retinal cell markers and examined by confocal microscopy. Antibody cone arrestin staining revealed cone cell body, inner and outer segments, axons, and cone pedicles.^45^^,^^50^ At P90, cone morphology was much better preserved in OSM-NP–treated eyes, with organized cell bodies and cone pedicles (Fig. 4C), in comparison with control retinas, where cone bodies and pedicles were disorganized (Fig. 4D). Among the secondary retinal neurons, rod bipolar cells connect photoreceptors and RGCs. As degeneration progresses, rod bipolar cells also undergo the secondary modification.^45^^,^^51^ The rod bipolar cells here, revealed by antibody protein kinase C-alpha, showed dense dendrites and axonal arborization in OSM-NP–injected eyes, whereas in the controls, bipolar cell dendrites and axonal terminals were dramatically decrease (Fig. 4E vs. Fig. 4F). Last, retinal sections from CNTF-NP and control treated eyes were stained with antibody against CNTF. A strong signal of CNTF was detected in the vitreous, RGC layer and some Müller glia cells and their end feet, whereas in control retina, the weak CNFT staining was localized to the RGC layer (Fig. 4G vs. Fig. 4H).

## Discussion

In this study, we demonstrated that sustained release of OSM and CNTF from formulated polysaccharide NP provided a neuroprotective effect on in vitro and in vivo models. Both complexes elicited significant prosurvival and pro-proliferation effects in iPSC-derived PPC and RGPC culture platforms; more important, the intravitreal delivery of both NP-trophic factors offered RGC protection in an ONC model of glaucoma, and photoreceptor and vision preservation into a rat model of RP. Specifically, significant vision preservation measured by ERG and OKR was observed in OSM-NP-treated RCS rats when compared with PBS-treated and untreated controls at 10 weeks after a single injection.

Previous studies on the effects of direct injection of OSM in S334ter-3 rats, the dominant RP model, demonstrated 50% photoreceptor preservation over a 1.5-week period.^24^^,^^25^ Here in the RCS rats, we demonstrated preservation of photoreceptors and retinal secondary neuron–bipolar cells with OSM-NP treatment extended over the course of 10 weeks. Correlated with morphologic preservation, significant visual function (from OKR-measured spatial visual acuity and ERG-measured light responsive electrical activity) was recorded in the retina in comparison with controls.

CNTF studies by comparison are more numerous in supporting its effect on preservation of photoreceptors and in experimental glaucoma models.^17^^,^^19^^,^^20^^,^^52^^–^^55^ These studies reported CNTF treatment can preserve photoreceptors, but also suppresses retinal electrophysiology.^30^^,^^56^ A recent study in a rhodopsin knockout mouse model of RP demonstrated that adeno-associated virus CNTF confers life-long protection against photoreceptor degeneration. Imaging of the visual cortex and assessment of visually evoked behavioral responses demonstrated that surviving cones retain function and signal correctly to the brain.^53^ The ERG suppression may relate to the CNTF concentration used. In the current study, we observe both CNTF-NP and OSM-NP preserve ERG response in the rodent model of RP compared with controls. Clinical trial studies show that CNTF treatment increased retinal thickness and slowed down the progression of vision loss, as determined by ERG^21^^,^^23^; however, a recent clinical study showed there is no effect over the long term (60–96 months),^31^ indicating that redosing may be needed for long-term efficacy.

The mechanisms underlying CNTF-mediated vision protection has been explored through transcriptome analysis in a study that reported a widespread upregulation of protease inhibitors, which may prevent cellular/extracellular matrix degradation and complement activation in retinal degenerative diseases.^53^ As a member of the IL-6 family of cytokines, OSM has homogenous protein sequence and structure as that of other family members.^57^ Studies in the mouse showed that human OSM activates the heterodimer of LIF receptor B and gp130, as does CNTF.^58^^,^^59^ An increase in STAT3 phosphorylation was localized to Müller cells after intravitreal delivery of OSM in an ONC model, indicating that the neuroprotective effect of OSM on the RGC is medicated by Müller cells.^26^ In the current study, both CNTF-NP and OSM-NP offered vision protection at P60 after a single intravitreal injection into rat models of retinal degeneration. The RPE cells in the RCS rats are unable to phagocytose photoreceptor outers segments owing to MERTK deletion. The mutant RPE cells retain some phagocytic activity either by MERTK-independent uptake or microglia-related phagocytosis.^60^ A previous study demonstrated that human umbilical tissue-derived cells can rescue phagocytic dysfunction in RCS RPE cells.^61^ A putative mechanism by which RPE phagocytosis might circumvent MERTK function is through the secretion of receptor tyrosine kinase ligands and opsonization of bridge molecules by human umbilical tissue-derived cells. In future studies, the mechanism by which these trophic factors in preserving photoreceptors in RCS rats will be studied.

Most of the animal studies using trophic factors were conducted at the early stage of retinal degeneration, including the current study. Future studies will also test whether this approach is still effective when applied at the middle or later stages of retinal degeneration. An encouraging study by Lipinski et al.^53^ showed that adeno-associated virus CNTF still offered long-term vision protection after dosing at a stage when rod photoreceptor degeneration was already advanced, mimicking the ocular phenotype of patients when presenting to the clinic. Another factor should be taken into consideration regarding late-stage intervention is the host retinal environment. It is well-established that there are secondary pathological modifications that occur as photoreceptors degenerate.^50^^,^^51^^,^^62^^,^^63^ The degenerative retinal environment may further compromise the function of preserved photoreceptors. Thus, combined treatments with trophic factors and agents that improve the retinal environment such as antioxidants and anti-inflammatory agents, may be needed for long-term vision protection at later stage of disease.

Another critical question that needs to be addressed is the NP-trophic factor release profile in vivo, so that targeted redosing can be performed to maintain vision protection for the long term. A previous clinical study of CNTF delivered by an intraocular encapsulated cell technology implant in patients with RP and geographic atrophy showed that the implants produced CNTF in the vitreous consistently over 2 years (the half-life of CNTF was 51 months^64^). However, monitoring trophic factor concentration from a vitreous sample is invasive and has a risk of complications. A recent study by Shpak et al.^65^ revealed that the CNTF concentration in the aqueous humor and lacrimal fluid showed a strong correlation. This method can, therefore, be used both in preclinical and clinical studies to measure CNTF or other agents noninvasively over time.

Like any drug, the toxicity of NPs is dependent on the route of administration and their systemic distribution. Once the NPs reach the blood circulation, they can accumulate in high perfusion organs such as liver, lungs, and kidneys. However, it is unlikely intravitreal injection of NPs will get into blood circulation because normally there are no blood vessels there. As for the NP used here, the backbone of the NP carrier, XDSCS NP, is made with DS and chitosan, which are both nontoxic biomaterials, biocompatible and bioresorbable polymers with safe safety profiles.^66^ However, we did observe NP aggregation in the vitreous around injection site, which could have further undermined the therapeutic effect by impeding global dispersal of trophic factors across the retina. The specific timelines of the NP degradation and potential toxicity need to be further investigated.

We have shown that OSM-NP and CNTF-NP are effective for sustained delivery of neurotrophic factors for neural protection. Future studies will depend on refined formulations. The OSM-NP and CNTF-NP used here were formulated in different batches, which may have created a technical variation. Synergistic studies combining OSM-NP and CNTF-NP may also be of interest as previous work has shown that CNTF in combination with brain-derived neurotrophic factor to promote significant photoreceptor preservation in in vitro mouse studies^66^ and combined trophic factor treatment also has been shown to offer long-term efficacy.^16^^,^^67^

The results of this study provide preliminary evidence in support of a potential clinical application for the long-term, sustained delivery of neurotrophic factors such as OSM and CNTF through a unique polysaccharide NP. Last, with the comparable therapeutic effect of OSM-NP and CNTF-NP in the RP rat model, this work offers a mutation-independent treatment strategy to benefit a diverse range of patients with RP and other retinal degenerative diseases, potentially including age-related macular degeneration and glaucoma.
